# Polyphasic identification (MALDI-TOF + ITS) of mucosal yeasts in hybrid marmosets from Rio de Janeiro

**DOI:** 10.1038/s41598-026-43653-y

**Published:** 2026-03-14

**Authors:** Debora Salgado Morgado, Gisela Lara Costa, Sócrates Fraga Costa-Neto, Iuri Veríssimo de Souza, Ricardo Moratelli, Manoel Marques Evangelista Oliveira

**Affiliations:** 1 Laboratory of Taxonomy, Biochemistry, and Bioprospecting of fungi, Oswaldo Cruz Institute, Oswaldo Cruz Foundation, Rio de Janeiro, Brazil; 2 Fiocruz Mata Atlântica, Oswaldo Cruz Foundation, Rio de Janeiro, Brazil

**Keywords:** Atlantic Forest, MALDI-TOF MS, Marmoset, Microbiota, Polyphasic taxonomy, Yeast, Diseases, Microbiology

## Abstract

*Callithrix* comprises primates popularly known as marmosets. In the city of Rio de Janeiro, the occurrence of a hybrid form of invasive species prevails. These animals, treated here as *Callithrix* spp., host several microorganisms in their microbiota, some of which can be pathogenic for humans. The aim of this study to describe culture-dependent yeast microbiota of the oral, rectal, and vaginal mucosae of hybrid marmosets (*Callithrix* spp.) inhabiting an urban–forest interface in the Atlantic Forest of Rio de Janeiro. Oral, rectal and vaginal samples were collected from 12 individuals during the winter of 2022. Animals were apparently healthy. The microbial agents obtained by culture isolation were identified to species level by polyphasic taxonomy using the MALDI-TOF MS and partial sequence of the internal transcribed spacer region (ITS1-5.8 S-ITS2) of ribosomal. A total of 26 fungal isolates were obtained. The most isolated species in the study was *Candida parapsilosis*, and the least frequent yeast were of genus *Pichia* sp., *Trichosporon* sp., and *Torulaspora* sp. Fungal infections in wild animals, depending on the causal agent, can be extremely pathogenic and contagious not only among animals, but also among humans, therefore fungal identification in these animals is important for future perspective.

## Introduction

In literature, studies on the microbiota of wild primates are scarce and regarding the fungal microbiota of marmosets^1,2^ these studies reported the presence filamentous fungi in the microbiota of these animals. Marmosets, genus *Callithrix*, comprise small primates popularly known as marmosets. Morphological and genetic evidence indicate that marmosets that occur in the city of Rio de Janeiro are hybrids of *C. jacchus* and *C. penicillata*^3^. Marmosets have several microorganisms in their microbiota, which can be pathogenic for us humans^2,4^ and they are in wide contact with humans in backyards, parks or walking along power lines in urban areas^5^.

There are few studies in the literature about microbiota in marmosets in the wild, especially those about yeasts^1,2^ which may hide important data on the transport of fungal agents to human and domestic animals’ health and emerging pathogenic fungi, which may pose a biological risk to humans, mainly in species that inhabit environments at the interface among urban and wild areas.

Due to factors such as climate change, emerging pathogens, such as *Candida auris*, an environmental yeast whose infection in humans is possibly attributed to global warming, are becoming increasingly common^6–9^. The specie *C. auris* is a public health problem in healthcare environments due to the high mortality rates associated with bloodstream infection^7,10^ and is remarkably resistant to antifungals and disinfectants^8^. Another yeast clinically important is *Candida parapsilosis*, which has shown increasing resistance to antifungal drugs in recent studies^11–15^. The emergence of fluconazole and echinocandin-resistant *C. parapsilosis* strains represents a significant challenge in clinical settings^16^. Furthermore, climate change may increase the geographic distribution of pathogenic species or their vectors, leading to the emergence of diseases in areas where they have not previously been described^17^. Therefore, this study aimed to describe the culture-dependent yeast microbiota of the oral, rectal, and vaginal mucosae of hybrid marmosets (*Callithrix* spp.) inhabiting an urban-forest interface in the Atlantic Forest of Rio de Janeiro, during the winter season, using a polyphasic taxonomic approach combining MALDI-TOF MS and ITS region sequencing.

## Methods

Hybrid marmosets (*Callithrix* spp.) were captured during the winter, in August 2022, during the pandemic period, and August of 2023, at Fiocruz Atlantic Forest Biological Station (Estação Biológica Fiocruz Mata Atlântica). The animals were captured in a peridomicile area, using Tomahawk traps (20 × 23 × 50 cm) installed on platforms attached to trees. In the field laboratory, the animals were anesthetized to collect vaginal, oral and rectal swabs following animal welfare standard. A uniform passage was carried out in the oral, rectal and vaginal cavities of the marmosets, with sterile swabs moistened with 0.9% saline solution and stored at a temperature of 4 °C until sample processing. Because they are considered invasive species in the city of Rio de Janeiro, marmosets undergo population biocontrol, during which the animals are removed from the wild and euthanized, in strict accordance with established ethical and institutional protocols.

Fieldwork was conducted under INEA license 012/2021 (SEI/ERJ – 15174767). The experimental protocol was approved by the Animal Ethics Committee of the Oswaldo Cruz Foundation (CEUA-Fiocruz; LW57-19). All procedures followed the guidelines of the American Society of Mammalogists^18^ and complied with Brazilian Law 11.794/08. Access to genetic material was registered in SISGEN (AAB9E36). Animals were anesthetized with ketamine (10–15 mg/kg), midazolam (0.1–0.2 mg/kg), acepromazine (0.04 mg/kg), and fentanyl (1.0 µg/kg) administered intramuscularly. Additional doses were provided as needed^19^. Animals remained under anesthesia only for the duration of the procedures. Euthanasia was performed under deep anesthesia by exsanguination following overdose of anesthetic agents or thiopental (200 mg/kg), via intraperitoneal or intracardiac injection, in accordance with Resolutions No. 714/2002, 876/2008, and 1000/2012 of the Conselho Federal de Medicina Veterinária. Death was confirmed by a licensed veterinarian.

For phenotypic characterization, Sabouraud Dextrose Agar (SDA; Difco, Becton-Dickinson and Company, USA), plus 400 mg/L of chloramphenicol and 25 mg/L of gentamicin were used. Swab samples collected from the oral, rectal, and vaginal cavities were directly streaked onto SDA places and incubated at 30 °C for 48 h. After incubation, the colonies were quantified as colony-forming units (CFU) according to Correa-Moreira and collaborators^20^, with modifications. Briefly, swab samples collected from the cavities were inoculated onto Petri dishes containing SDA and incubated at 30 °C for 48 h. Colonies displaying macroscopic morphoogies (based on color, texture, and margin characteristics) were selected and subculture onto fresh SDA plates to obtain pure isolates for further macroscopic characterization^20^. Due to the low number of colony-forming units (CFU) observed from the swab plating onto SDA plates, serial dilution was not required. The number of colonies observed on the agar plates remained within the ideal countable range (30 to 300 CFU per plate), allowing for reliable quantification^21^. For chromogenic differentiation, a fresh yeast colony was suspended in sterile 0.9% saline solution and adjusted to McFarland standard n° 1, using visual turbidity comparison. An aliquot of 1 l of this suspension was inoculated onto CHROMagar^®^ *Candida* plates (Difco, Becton-Dickinson and Company, USA) and incubated at 37 °C for 48 h. The results were interpreted based on manufacturers guidelines and used as a presumptive identification method, as follows: *Candida albicans* - colonies light green to medium green, *Candida tropicalis* - blue-gray, bluish gray to blue-green or metallic blue colonies with or without violet halos in the middle, *Candida krusei* - large flat colonies, light pink to light red with a whitish border, and nonspecific nuclei, other species. For micromorphological characterization was performed using Corn Meal Agar (ThermoFisher Scientific, Waltham, MA, USA). Plates were incubated at 30 °C for 48 h and examined for the presence of pseudohyphae and blastoconidia, as previously described^22^.

The microbial isolates obtained by culture were identified to species level using polyphasic taxonomic approach. Matrix-Assisted Laser Desorption/Ionization Time-of-Flight Mass Spectrometry (MALDI-TOF MS) was employed as a rapid screening tool. Mass spectra were generated using a Microflex mass spectrometer and analyzed with MALDI Biotyper software version 3.1 (Bruker Daltonics), as previously described Pinto and collaborators^23^. All isolated were further confirmed by sequencing of the internal trascribed sapcer (ITS) region, which was considered the gold standard for species-level identification.

All strains obtained for partial sequencing of the internal transcribed spacer region (ITS1-5.8 S-ITS2) region of the ribosomal DNA using colony PCR (Polimerase Chain Reaction^24^. Briefly, yeats were grown on SDA plates at 30 °C for 48 h. A small portion of a single colony was used as template for PCR amplification. Subsequently, amplification was performed using 25ng of genomic DNA obtained in a final volume of a 50 µl reaction volume, using 10 pmol of universal fungal primers ITS1 (CGTAGGTGAACCTGCGG) and ITS4 (TCCTCCGCTTATTGATATGC). The QIAquick^®^ PCR Purification Kit (QIAGEN^®^) was used to purify the amplified product, following the manufactuer’s instructions. Sequencing was carried out at the Sequencing Platform at Fundação Oswaldo Cruz - PDTIS/FIOCRUZ, Brazil. Chromatograms were edited using the CodonCode Aligner software 11.3 and consensus sequences were compared with reference sequences available in the NCBI/GenBank database using BLASTn 1.4.0 (Basic Local Alignment Search Tool). Phylogenetic analysis was performed using the Neighbor-Joining method of^25^ with 1000 replicate bootstraps.

For statistical analysis, Fisher’s exact test was used to assess the association between categorical variables, sex, and cavity. A p-value < 0.05 indicates significant associations in the statistical tests. The analysis was performed using R 4.5.2 software.

## Results

**Fig. 1:**
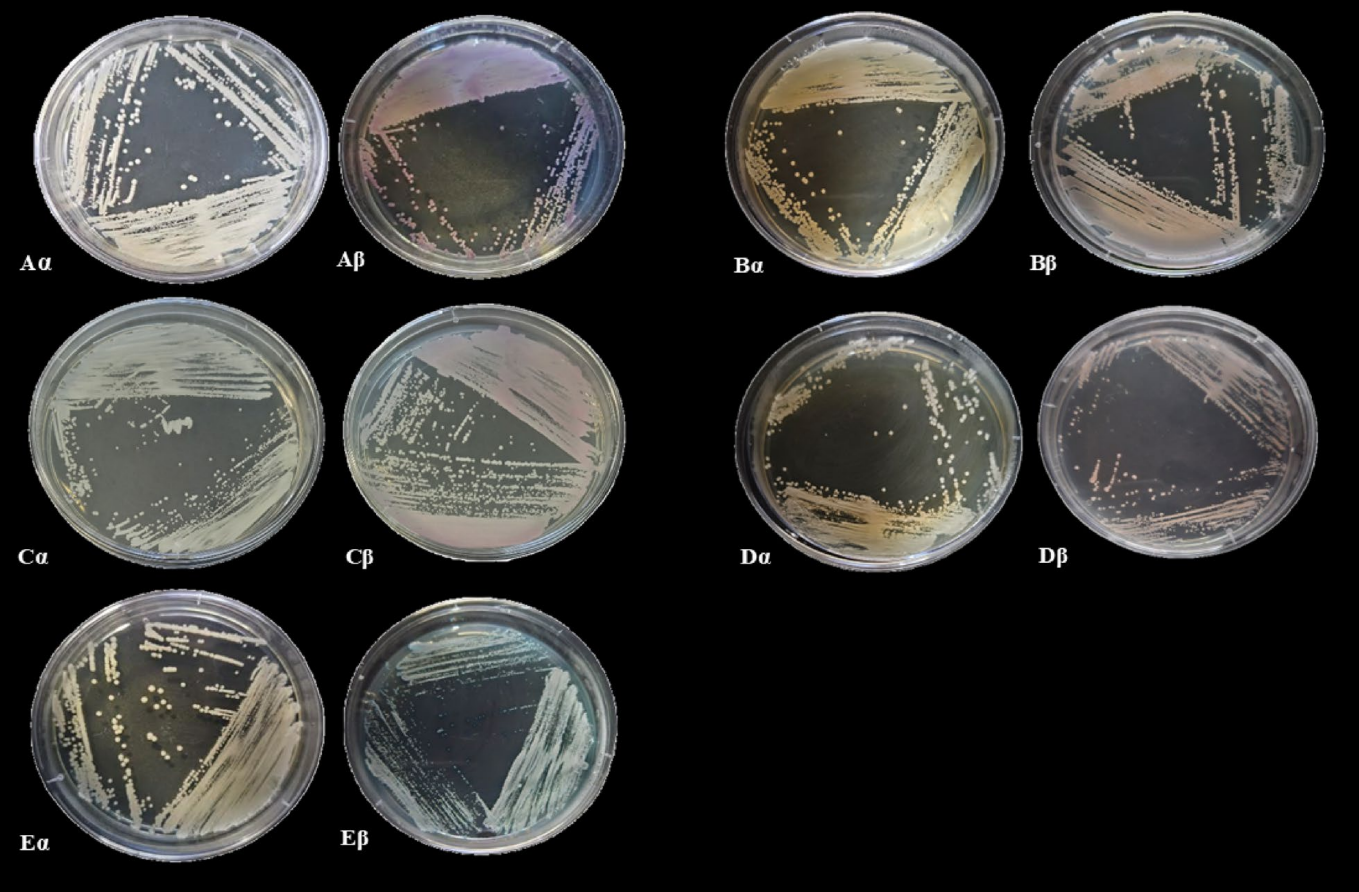
Viewing species on *Sabouraud Dextrose Agar* (SDA) (α) and CHROMagar^®^ *Candida* (β) 37 °C for 48 h, respectively. **Aα**,** Aβ**. IOC/1061 SO (*Pichia myanmarensis*); **Bα**,** Bβ.** IOC/1063 SO (*Candida parapsilosis*); **Cα**,** Cβ.** IOC/1065 SR (*Pichia manshurica*); **Dα**,** Dβ**. IOC/1063/1 SV (*Torulaspora pretoriensis*); **Eα**,** Eβ**. IOC/1063/2 SV (*Trichosporon asahii*).

In the first fieldwork (winter of 2022), the day was cold and rainy (22 °C), resulting in a total of 5 animals being captured. In total, 10 yeast strains were isolated from this field, the cavity that showed the greatest fungal growth was the vaginal cavity. Table 1 shows the number of CFU per cavity, obtained from swab collection (Table 1). In the second fieldwork (winter of 2023), the weather was clear (25 °C). A total of 7 animals were captured and all clinical samples obtained of the cavities showed yeast-like growth white and smooth in SDA medium (Fig. 1A-Eα), being isolated 16 strains.

**Table 1:** Table of colony-forming unit (CFU/Swab) of fungi isolated from the cavities of hybrid marmoset (*Callithrix* spp.) captured in the Atlantic Forest, Rio de Janeiro.

Year	Animal	Sex	CFU/Swab		
			Oral	Rectal	Vaginal
Winter 2022	FMA 1061	Male	20	0	NA
	FMA 1062	Male	16	0	NA
	FMA 1063	Female	33	0	45
	FMA 1064	Male	20	33	NA
	FMA 1065	Female	0	20	28
Winter 2023	FMA 1746	Female	30	35	28
	FMA 1747	Female	30	35	30
	FMA 1748	Male	40	20	NA
	FMA 1749	Male	20	40	NA
	FMA 1750	Female	30	35	28
	FMA 1751	Male	0	25	NA
	FMA 1753	Male	25	30	NA
	NA: Male				

The marmosets were identified as hybrids of (referred here as *Callithrix* spp.) based on external traits. A total of 7 males and 5 females were collected, all non-pregnant. In some samples of cavities that did not show growth of yeast (Table 1).

No significant difference in oral or rectal prevalence was observed between males and females (Fisher’s exact test, *p* = 1.0 for both comparisons). Vaginal samples were obtained only from females; therefore, no statistical comparison by sex was possible for this cavity (Table 2).

**Table 2:** Distribution of positive and negative results from oral, rectal, and vaginal samples of male and female marmosets (*Callithrix* spp.).

Variable		Female	Male
Oral	Positive	4	6
	Negative	1	1
Rectal	Positive	4	5
	Negative	1	2
Vaginal	Positive	5	NA
	Negative	0	NA
NA: Male			

In total, 26 yeast isolates were obtained from 12 animals, and these yeasts have grown from the oral, vaginal and rectal regions of the marmosets (Table 3).

**Table 3:** Table of polyphasic taxonomic, characterization phenotypic and characterization molecular of fungi isolated from hybrid marmoset (*Callithrix* spp.) captured in the Atlantic Forest, Rio de Janeiro.

Strain	Characterization phenotipic	Characterization molecular		
	Presumptive species	MALDI-TOF (score)	Sequencing	GenBank
IOC/1061 SO	*Candida* spp.	*Pichia myanmarensis* (1.82)	*Pichia myanmarensis*	PV870192
IOC/1062 SO	*Candida* spp.	*Pichia myanmarensis* (1.85)	*Pichia myanmarensis*	PV870191
IOC/1063 SO	*Candida parapsilosis*	*Candida parapsilosis* (1.98)	*Candida parapsilosis*	PV866880
IOC/1063/1SV	*Candida* spp.	*Torulaspora* spp. (1.45)	*Torulaspora pretoriensis*	PV870202
IOC/1063/2SV	*Candida tropicalis*	*Trichosporon asahii* (1.94)	*Trichosporon asahii*	PV870156
IOC/1063/3SV	*Candida parapsilosis*	*Candida parapsilosis* (1.86)	*Candida parapsilosis*	PV866881
IOC/1064 SO	*Candida parapsilosis*	*Candida parapsilosis* (1.80)	*Candida parapsilosis*	PV866882
IOC/1064 SR	*Candida parapsilosis*	*Candida parapsilosis* (1.87)	*Candida parapsilosis*	PV870144
IOC/1065 SR	*Candida* spp.	*Pichia manshurica* (2.09)	*Pichia manshurica*	PV870190
IOC/1065 SV	*Candida parapsilosis*	*Candida parapsilosis* (1.99)	*Candida parapsilosis*	PV870145
IOC/1746 SO	*Candida parapsilosis*	*Candida parapsilosis* (1.94)	*Candida parapsilosis*	PV870147
IOC/1746 SV	*Candida parapsilosis*	*Candida parapsilosis (*1.94)	*Candida parapsilosis*	PV870148
IOC/1746 SR	*Candida parapsilosis*	*Candida parapsilosis* (2.05)	*Candida parapsilosis*	PV870146
IOC/1747 SO	*Candida parapsilosis*	*Candida parapsilosis* (1.85)	*Candida parapsilosis*	PV866879
IOC/1747 SV	*Candida parapsilosis*	*Candida parapsilosis* (2.06)	*Candida parapsilosis*	PV866884
IOC/1747 SR	*Candida parapsilosis*	*Candida parapsilosis* (2.14)	*Candida parapsilosis*	PV866885
IOC/1748 SO	*Candida parapsilosis*	*Candida parapsilosis* (1.80)	*Candida parapsilosis*	PV870150
IOC/1748 SR	*Candida parapsilosis*	*Candida parapsilosis* (1.87)	*Candida parapsilosis*	PV870149
IOC/1749 SO	*Candida parapsilosis*	*Candida parapsilosis* (2.08)	*Candida parapsilosis*	PV866887
IOC/1749 SR	*Candida parapsilosis*	*Candida parapsilosis* (2.06)	*Candida parapsilosis*	PV866883
IOC/1750 SO	*Candida parapsilosis*	*Candida parapsilosis* (1.86)	*Candida parapsilosis*	PV870152
IOC/1750 SR	*Candida parapsilosis*	*Candida parapsilosis* (1.88)	*Candida parapsilosis*	PV866886
IOC/1750 SV	*Candida parapsilosis*	*Candida parapsilosis* (1.88)	*Candida parapsilosis*	PV870151
IOC/1751 SR	*Candida parapsilosis*	*Candida parapsilosis* (1.99)	*Candida parapsilosis*	PV870153
IOC/1753 SO	*Candida parapsilosis*	*Candida parapsilosis* (1.82)	*Candida parapsilosis*	PV870154
IOC/1753 SR	*Candida parapsilosis*	*Candida parapsilosis* (1.80)	*Candida parapsilosis*	PV870155

SO: Oral swab; SR: Rectal swab; SV: Vaginal swab/Score: 1,7 − 1,99: genus-level; 2,0: spcies-level.

Through phenotypic characterization, macroscopic aspects of the colonies on SDA and CHROMagar *Candida* medium, we identified 21 strains exhibiting a white to pink appearance on CHROMagar *Candida*, consistent with the profile of *Candida parapsilosis* (Fig. 1Bβ). 3 strains with indeterminate profile due to color variation, identifying as *Candida* spp., (Fig. 1 Aβ, C-Dβ), and 1 strain exhibiting a with blue appearance on CHROMagar *Candida*, consistent with the profile of *Candida tropicalis* (Fig. 1Eβ). The IOC/1063/2 SV strain was the only one showed growth on SDA with colonies cream with a rough appearance (Fig. 1Eα).

**Fig. 2:**
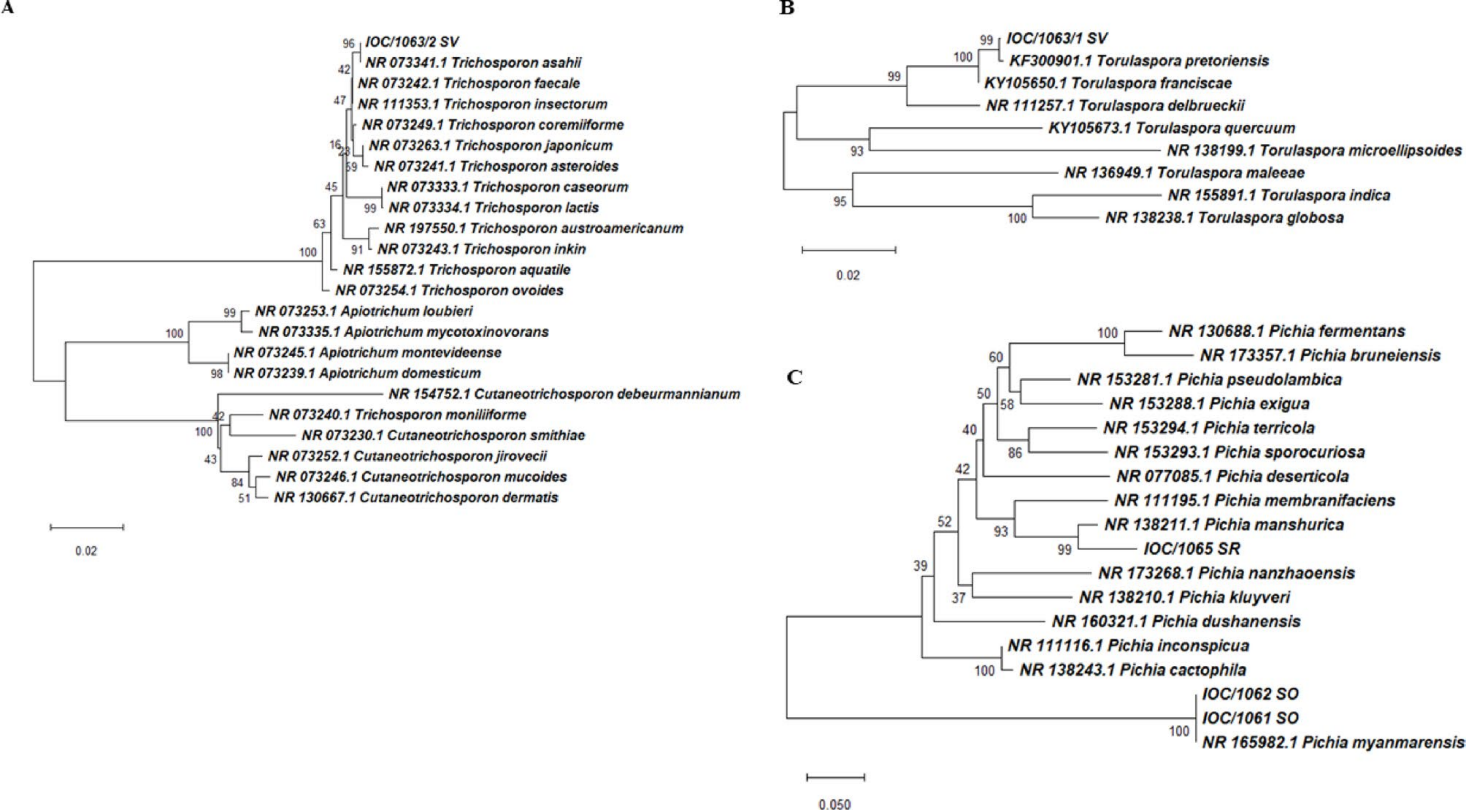
Phylogenetic trees of the yeasts identified by sequencing of the ITS region. **A**: *Trichosporon* spp.: this analysis involved 23 nucleotide sequences, and a total of 549 positions were obtained in the final dataset. **B**: *Torulaspora* spp.: this analysis involved 9 nucleotide sequences, and a total of 1050 positions were obtained in the final dataset. **C**: *Pichia* spp.: this analysis involved 18 nucleotide sequences, and a total of 729 positions were obtained in the final dataset. Phylogenetic analyses were conducted in MEGA4 according to Tamura-Nei^46^.

**Fig. 3:**
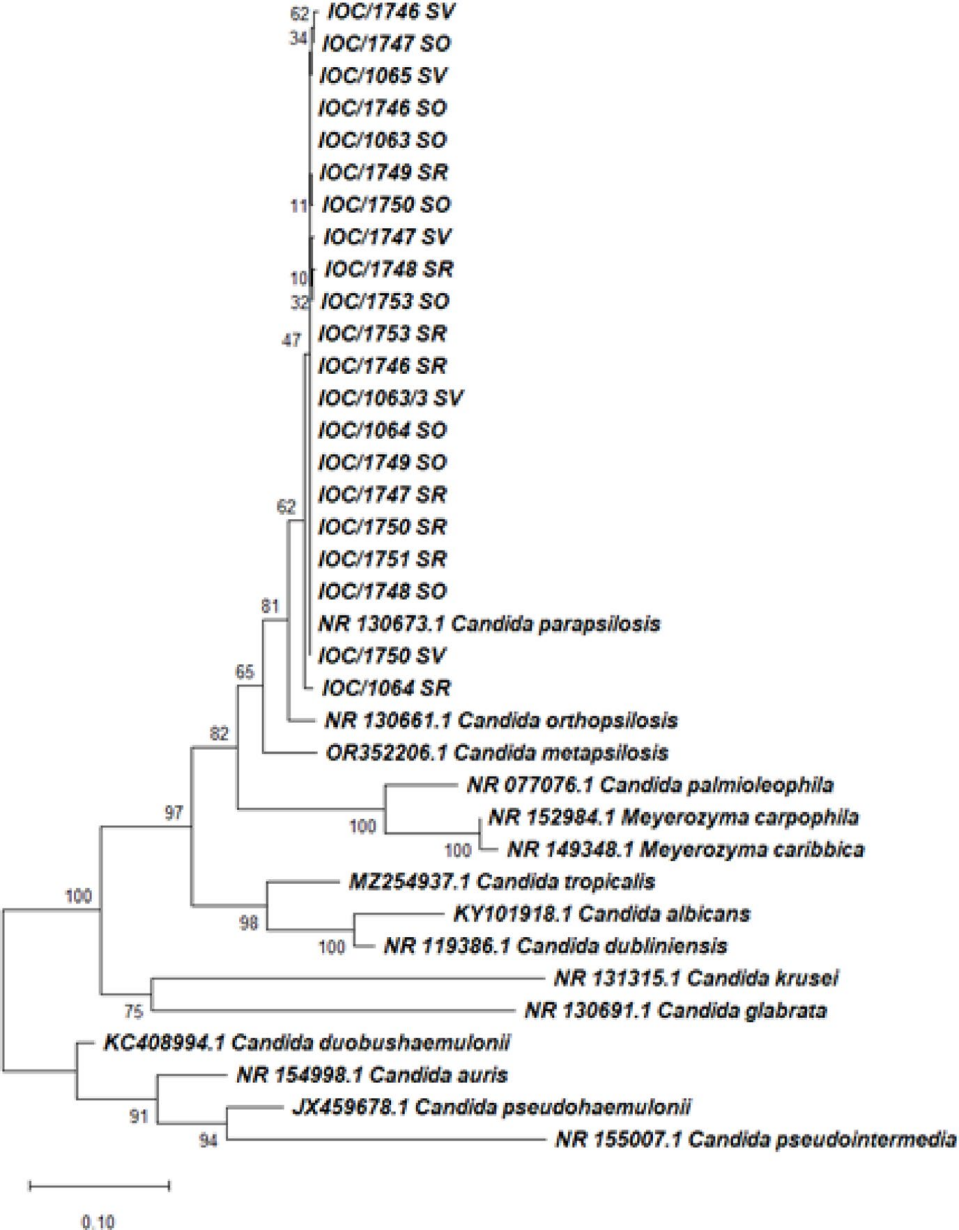
Phylogenetic trees of *Candida* spp. identified by ITS region sequencing This analysis involved 36 nucleotide sequences, and a total of 883 positions were obtained in the final dataset. Phylogenetic analyses were conducted in MEGA4 according to Tamura-Nei^46^.

Identification by MALDI-TOF MS *C. parapsilosis* (21), *Pichia myanmarensis* (2), *Pichia manshurica* (1) *Torulaspora spp.* (1) and *Thrichosporon asahii* (1) (Table 3). Applied the polyphasic taxonomy in ITS region sequencing twenty-one strains were of species *C. parapsilosis*, *P. myanmarensis* (2), *P. manshurica* (1), *Torulaspora preteoriensis* (1), and *T. asahii* (1) (Figs. 2 and 3). All sequences were compared to available ITS sequences from the NCBI/GenBank database with reference strains. In relation to BLAST analysis, the sequences exhibited 98–100% coverage/identity of species *C. parapsilosis* (LC390063.1; OM523851.1; KJ734200.1); *P. manshurica* (OR554067.1); *P. myanmarensis* (OW988376.1); *T. pretoriensis* (KF300901.1); *T. asahii* (KY105711.1). All sequences were deposited in the database GenBank under accession numbers PV866879- PV866887; PV870144-PV870156; PV870190-PV870192 (Table 3).

In micromorphology on Corn Meal agar (ThermoFisher Scientific, Waltham, MA, USA), the presence of pseudo-hyphae and blastoconidia was visualized in all strains of *C. parapsilosis*. The strains IOC/1061 SO, (*P. myanmarensi*), IOC/1062 SO (*P. myanmarensi*), IOC/1063/1 SV (*T. pretoriensis*), and IOC/1065 SR (*P. manshurica*) showed blastoconidia and not showed pseudo-hyphae. The strain IOC/1063/2 SV (*T. asahii*) showed blastoconidia, and hyphae.

In our study, MALDI-TOF MS was used as a rapid screening method to identify the samples. The strain IOC/1063/1 SV, was the only one for which MALDI-TOF MS provided a divergent species-level identification, identifying as *Torulaspora* spp., identification at gender level due to low score, according to Cassagne and collaborators^26^, whereas ITS sequencing confirmed it as *Torulaspora pretoriensis*.

According to polyphasic taxonomy, the most isolated species was *C. parapsilosis* and subsequent to performing molecular analysis by MALDI-TOF MS and ITS sequencing, the most frequently identified species was *C. parapsilosis*.

## Discussion

To date, the literature indicates that these species of the *Candida parapsilosis*, *Trichosporon asahii*, *Torulaspora pretoriensis*, *Pichia manshurica* and *Pichia myanmarensis*, have not been isolated in marmosets to date. In *Leontopithecus chrysopygus*, the black lion tamarin, the genera *Candida* and *Trichosporon* have already been isolated in the literature^27^.

According to polyphasic taxonomy, we observed in our study that the phenotypic characterization of *C. parapsilosis* samples presented distinct macromorphologies in culture, with variations in shades from pink to white, with only one species identified from the *C. parapsilosis* complex identified in the study.

The MALDI-TOF MS is a viable alternative for identifying the yeast microbiota of marmosets, despite the divergent identification at the species level of the genus *Torulaspora*. Previous studies^28,29^ identified *T. delbrueckii* by MALDI-TOF MS. However, to date, we have not found confirmation in the literature by MALDI-TOF MS identifying the yeast *T. pretoriensis*, isolated in our study. And according to Augustine et al.^30^, more than 30% of environmental yeasts were not identified by MALDI-TOF MS because the strains were not included in the database. Therefore, further studies are needed to better identify this genus by MALDI-TOF MS.

Climate change and biodiversity loss, associated with factors such as deforestation, wildfires, and agricultural activities, are important drivers of the emergence of wildlife-associated diseases^31^. From a One Health perspective, our findings contribute to the understanding of the diversity of yeasts present in *Callithrix* spp., considering that marmosets are primates that frequently inhabit areas close to urban settings and human populations, often occupying power lines, public squares, and residential backyards^5^. Furthermore, the proximity of these primates to urbanized areas creates ecological interfaces that may facilitate the exchange of microorganisms among wildlife, the environment, and humans. These findings reinforce the importance of monitoring the fungal microbiota of wild animals that occupy anthropized environments, especially in scenarios of rapid environmental change. Although many of these yeasts are part of the environmental microbiota, such as *T. pretoriensis*, *P. manshurica*, and *P. myanmaresnis*, some species may act as opportunistic pathogens, mainly in immunocompromised individuals, such as *C. parapsilosis*^32^.

The most frequent species in the study, *C. parapsilosis*, is a commensal yeast found on the skin and mucous membranes of humans^32^. It has been isolated from domestic animals, insects, soil and marine environments^32^. Belonging to the complex comprising three distinct species: *Candida parapsilosis* sensu stricto, *Candida orthopsilosis* and *Candida metapsilosis*. It is a yeast of concern for immunocompromised patients and hospital areas due to its infection in catheters and underweight neonates^32^. The yeast, *C. parapsilosis* exhibits resistance to echinocandins and multidrug resistance, highlighting its importance as an emerging threat to global health^32,33^.

The species *C. parapsilosis* is a yeast considered emerging, due to its ability to form biofilms in catheters, it can cause outbreaks in hospitals, in addition to cross-contamination due to the hands of health professionals, contaminated material and, mainly, due to the formation of biofilms, it increases resistance to antifungal agents and impairs the host’s immune response^32,33^. Species of the genus *Candida* are commonly isolated from the skin and mucosal surfaces of healthy dogs and cats; however, they can become pathogenic when the host’s immune defenses are compromised^33^. According to the literature on the microbiota of animals, they identified that *C. parapsilosis* showed resistance to fluconazole and these strains produced more biofilm when compared to human strains^33,34^. In our study, *C. parapsilosis* was identified in all three regions of microbiota, oral, rectal and vaginal, of healthy marmosets.

Another emerging pathogen isolated was yeast species *T. asahii*, these yeasts are just behind the genus *Candida* in pathogenicity^35^. The yeast *T. asahii* recovered from the vaginal microbiota of a marmoset. Previous studies have reported in soil, mammals, birds and pigeon excrement^36^. Its clinical aspect is considered rare, but in immuno-compromised patients it is considered fatal^35^. And is considered an emerging global pathogen associated with invasive fungal infections^36^. Regarding resistance, *Trichosporon* species are intrinsically resistant to echinocandins and show low in vitro susceptibility to amphotericin B^36^. Additionally, cases of *T. asahii* with limited susceptibility to antifungal classes have been reported. Currently, fluconazole and voriconazole are the main antifungal agents used in clinical practice, however, clinical isolates with reduced susceptibility to both triazoles have already been identified^36^.

In addition to emerging pathogens yeast, we also identified environmental yeasts, such as the *P. myanmarensi* and *P. manshurica*, which are found in various fermenting foods, such as table olives, cocoa beans, wine and vinegar^37^. And yeast *T. pretoriensis*, which has already been isolated from soil, orange, wines and invertebrates^38^. Due to the marmoset’s diet, eating fruits, plant exudates, and insects, it can influence the prevalence of yeasts present in its microbiota^39,40^, we identified environmental yeasts such as *P. myanmarensis*, *P. manchurica* and *T. pretoriensis*, which are environmental yeasts found in fruits and soil.

Global warming affects the environment as a whole, without distinction between humans and animals. Due to anthropogenic activities, gas emissions like carbon dioxide, methane gas and nitrous oxide are causing an increase in the temperature of the planet^41^. Climate change occurs causing natural catastrophes, impacting men and animals.

During both capture periods, the temperature was within the expected for winter (22°−25 °C). In the southeastern region of Brazil, the study site, the mean temperature was 22.8 °C in the winter of 2022 and 24.9 °C in the winter of 2023, indicating an increase of 2.1 °C in the period of one year^42^. However, it is known that the temperature of our planet is increasing more and more, as the climate mitigation report AR6 stated that the policies in place in 2020 would not prevent emissions from continuing to increase, causing a warming of 3.2 °C by 2100^41,42^. Consequently, one hypothesis is that, increased temperatures, humidity and sun exposure, may enhance the growth of fungi and other microorganisms in the animal’s microbiota^43^.

The behavior of wild animals is affected by temperature, cold, heat, rain, and wind. Temperature influences the amount of time primates spend on the ground versus in the trees^44^. Arboreal activity tends to increase when fruit is available. If fruit trees are spatially clustered, primates may need to travel longer distances to obtain food. Terrestrial activity, in turn, may vary according to temperature, humidity, and precipitation^44^. These factors may partially explain the low yeast diversity detected in these animals. The sampling day was marked by cloudy weather and heavy rainfall in the Atlantic Forest, which may have influenced fungal detection. Nonetheless, additional studies with larger sample sizes and sampling across multiple seasons are required to confirm this observation, as one of the main limitations of our study was the restriction to a single season. In addition, the reliance on culture-based methods may have limited the detection of non-cultivable or low-abundance fungal species. Therefore, future research should focus on characterizing the yeast microbiota of marmosets across different seasons, as well as increasing the number of animals sampled and incorporating complementary molecular approaches.

In the literature, studies on the microbiota of free-ranging primates are scarce. Regarding the fungal microbiota of marmosets^1,2^. Our results corroborate the literature, where the majority of the isolates were environmental yeasts, as these yeasts are present in the soil, fruits and leaves. There was no significant difference between the sexes of the marmosets and the species of yeast isolated. Being similar to that found in the microbiota of filamentous fungi in marmoset^2^.

Marmosets are animals considered beloved by humans, as a result of which these animals were dispersed from their place of origin, the Brazilian northeast, being taken by truck drivers as pets to Rio de Janeiro. However, marmosets are wild animals and due to their behavior, they are considered unpleasant. Marmosets were abandoned in the forests close to urban areas, which is why we found these animals on wire posts, squares and in houses^5^.

Marmosets have several microorganisms in their microbiota, which can be pathogenic for us humans. In addition to being transmitters of rabies^4^. In Brazil, in the state of Pernambuco, a 56-year-old woman died from the rabies virus after being bitten by a marmoset. Family members reported that, to protect her 3-year-old grandson, the patient tried to push the animal away and was bitten on the finger. The patient sought medical attention but did not receive anti-rabies prophylaxis^45^.

Although marmosets are considered tourist attractions in many cities to this day, due to their charismatic appearance, wild animals exhibit territorial behavior and may feel threatened by humans when taking photos or feeding them. Therefore, it is not recommended to feed or handle them unless you are a qualified professional.

Fungal infections in wild animals, depending on the causal agent, can be extremely pathogenic and contagious, both for animals and humans^2,4^. However, in our study, all marmosets were healthy at the time of capture, without lesions by fungi. According to the literature^1,2^, the prevalence of pathogens present in healthy free-living animals is rarely reported. The presence of emerging yeast in the microbiota of marmosets suggests their potential as sentinel models for monitoring clinically important fungal agents. Marmosets, being exposed to anthropogenic environments and sharing ecosystems with humans, could serve as important bioindicators of emerging yeast. According to the one health principle, knowing the fungal microbiota of wild animals, such as marmosets, is important to anticipate risks to human, animal and environmental health, and thus carry out preventive actions when necessary.

## Conclusions

It is worth highlighting, the most frequent species in the study was *Candida parapsilosis* the emerging pathogen and we reported for the first time *C. parapsilosis*, *Trichosporon asahii*, *Torulaspora pretoriensis*,* Pichia manshurica*, and *Pichia myanmaresnis* in marmosets. The winter of 2023, which was 2.1 °C warmer compared to the previous winter, showed an increase in the growth of yeasts in higher temperatures. Further studies are needed to better understand the presence and seasonal distribution of emerging pathogenic fungi in these wild animals, as well as to increase the sample size. These efforts will contribute to a better undestanding of the ecological role and potencial zoonotic risks associated with fungal species in wildlife. In addition to reinforce that wild animals should not be treated as domestic pets, nor should they be fed by humans. This may increase the risk of zoonotic transmission of infections, including emerging pathogenic fungi also that proote change of yeast microbiota in these animals. Therefore, conducting fungal microbiota screening in free-living marmosets is essential to prevent future epidemiological outbreaks and to protect human, animals, and environmental health, in accordance with One Health.

## Data Availability

All sequences were deposited in the database GenBank under accession numbers PV866879- PV866887; PV870144-PV870156; PV870190-PV870192.
